# Impacts of sulfamethoxazole stress on vegetable growth and rhizosphere bacteria and the corresponding mitigation mechanism

**DOI:** 10.3389/fbioe.2024.1303670

**Published:** 2024-02-08

**Authors:** Jiawei Ren, Hongbin Lu, Shaoyong Lu, Zhanggen Huang

**Affiliations:** ^1^ State Key Laboratory of Environmental Criteria and Risk Assessment, National Engineering Laboratory for Lake Pollution Control and Ecological Restoration, Chinese Research Academy of Environmental Sciences, Beijing, China; ^2^ Lake Forest Academy, Lake Forest, IL, United States; ^3^ State Key Laboratory of Coal Conversion, Institute of Coal Chemistry, Chinese Academy of Sciences, Taiyuan, China

**Keywords:** sulfamethoxazole, basil, cilantro, spinach, antibiotic resistance

## Abstract

Antibiotics are an important pharmaceutical class excessively used by humans. Its presence in the soil can impact plant growth and induce antibiotic resistance. This research studies the effect of sulfamethoxazole (SMX) on plant growth, rhizosphere bacteria composition, and resistance genes. Two sets of vegetables (basil, cilantro, and spinach) were treated separately with water and SMX solution. The plant growth data and soil samples were collected and analyzed. The results revealed that SMX increased spinach leaf length (34.0%) while having no significant impacts on basil and cilantro. On the other hand, SMX improved the bacterial diversity in all samples. The shifts in the abundance of plant growth-promoting bacteria could indirectly affect vegetable stem and leaf length. SMX also significantly increased the abundance of resistance genes *Sul1* and *Sul2*. A further study into the correlation between bacteria highlights the importance of *Shingomonas* and *Alfipia* for inhibiting the spread of key resistance gene hosts, namely, *Pseudomonas*, *Stenotrophomonas*, and *Agrobacterium*. This research provides insight into SMX’s impact on vegetable growth and microbial diversity. It also points out important microbial interactions that could potentially be utilized to mitigate ARG proliferation.

## 1 Introduction

Antibiotics have been widely used for medication and animal husbandry (Patangia et al., 2022). They are the most important pharmaceutical class today and their consumption has increased by 46% between 2000 and 2018 (Browne et al., 2021; Uddin et al., 2021). However, antibiotics are not completely metabolized by humans and animals. As much as 30%–90% of antibiotics are excreted through urine and feces (Zhang et al., 2015). They end up in the environment through runoffs, manure treatments, and effluents of wastewater plants (Zhao et al., 2020).

Sulfamethoxazole (SMX) belongs to a group of antibiotics called sulfonamide (SA) (Coleman, 2022). Because of its affordability and versatility, SA has been commonly applied and is the second most concentrated antibiotic in the soil (approximately 25 ppm) (Robles-Jimenez et al., 2021). SA can affect plant growth and ultimately threaten public health through the food chain (Baran et al., 2011). SMX in particular has caused antibiotic resistance in the past decade worldwide (Du et al., 2019; Wang et al., 2019; Chia-Wei et al., 2022). Antibiotic-resistant genes (ARGs) are the main reasons that bacteria develop antibiotic resistance (Zhao et al., 2020). Antibiotic presence in the soil induces ARGs by exerting selective pressures. Bacteria develop resistance using mechanisms that tolerate, extrude, interfere, inactivate, or destruct the antibiotics. Those modifications are facilitated by mutational resistance and passed between bacterial species through horizontal gene transfer (Skandalis et al., 2021). ARB can be transmitted from farms to the human body via the food chain and environment, which causes prolonged illness, increased hospitalization, and treatment failure (World Health Organization). Even if ARB is killed or damaged, ARGs can be released into the water, soil, or air and transferred into other bacteria (Zhang et al., 2020; Zhuang et al., 2021). The World Health Organization considers antibiotic resistance to be one of the most urgent public health concerns (World Health Organization, 2023). According to a United Nations report, in 2019 an estimated 1.27 million global deaths were directly caused by drug-resistant disease (United Nations Environmental Programme, 2023).

Because of the limited access to clean water, wastewater is being increasingly used and recycled in crop irrigation worldwide (Slobodiuk et al., 2021). However, due to insufficient treatment, this practice exposes crops to antibiotics in the wastewater and affects plant growth (Kampouris et al., 2021). Pan and Chu’s study in Hong Kong found that the presence of sulfamethazine (157 mg/L) can significantly inhibit lettuce root and stem elongation (Pan and Chu, 2016). Several studies have shown that high antibiotic concentrations can lead to delayed germination, reduced root and stem length, decreased biomass, and even death among crops. Most of the research used starchy crops (maze, wheat, pea, etc.) or fruity crops such as cucumbers.

Not many studies have been done with vegetables such as spinach, cilantro, and basil (Migliore et al., 1995; Liu et al., 2009; Yang et al., 2010; Michelini et al., 2012). Therefore, investigating the effect of common antibiotics on vegetables could be valuable to modern agricultural development. Considering the connection between soil antibiotics, plants, and public health, it is crucial to understand how antibiotics impact vegetable growth, soil bacteria composition, and ARGs. To simulate the effects of SMX in wastewater irrigation, in this research three vegetables (spinach, cilantro, and basil) were treated with SMX solution. The plant growth analysis revealed the vegetable’s physiological response to SMX. The metagenomic analysis explored changes under SMX stress on rhizosphere bacteria and ARGs. These results provide a reference for SMX’s impact on farmland through wastewater irrigation and also offer suggestions to mitigate ARGs.

## 2 Materials and methods

### 2.1 Site setup

Two sets of ten pots were installed. One set was treated with sulfamethoxazole solution (75 mg/L) and the other with water. Within each set, three pots were assigned to each vegetable (spinach as *Spinacia oleracea*, cilantro as *Coriandrum sativum*, and basil as *Ocimum basilicum*), and one pot was designated for soil as control. All pots (22 cm in diameter, 21 cm in height) were filled with a layer of 18 cm organic soil as the substrate for plant growth. SMX solution (75 mg/L) was prepared using 98% SMX from Shanghai Aladdin Biochemical Technology Co., Ltd. The experiment was conducted under a temperature range of 27°C–33°C. Two sets of pots were treated with respective solutions (avg. 60mL/day) to keep them moist in an environment with a relative humidity of 57%.

The spinach seeds were directly dispersed into the pots. But basil and cilantro seeds had to break dormancy for all plants to sprout at a relatively similar time. Each cilantro seed was a pod of two or three individual seeds. The pods were broken gently using the handle of a metal spoon and soaked in 38°C water for 24 h. The basil seeds were placed on a sheet of moist paper towels for 24 h. After the pre-germination process, 12 seeds were dispersed evenly in each pot, different by plant type. The seeds were covered with an additional 3 mm layer of soil. Shortened names are assigned to each of the eight experimental groups (Table 1).

**TABLE 1:** Group name with corresponding treatment and vegetable.

Group name	Treatment		Vegetable			
	SMX	Water	Soil	Basil	Cilantro	Spinach
SOA	**×**		**×**			
BAA	**×**			**×**		
CIA	**×**				**×**	
SPA	**×**					**×**
SOT		**×**	**×**			
BAT		**×**		**×**		
CIT		**×**			**×**	
SPT		**×**				**×**

### 2.2 Growth data and soil sample collection

After 19 days, four plants of medium height in each group were selected to track height and leaf growth. Heights were measured from the surface of the soil to the highest point of the plant. An original soil sample was collected. Three weeks after sowing, soil samples were collected from each group by either gathering soil of the plant rhizosphere or if no plant was present they were directly taken from the pot. All samples were kept at −80°C before being processed and analyzed at Wekemo Tech Co., Ltd. Shenzhen, China.

### 2.3 DNA extraction

DNA was extracted from the soil samples using the CTAB method. First, the soil samples were transferred into 2.0-mL Eppendorf tubes containing 1,000 µL of CTAB extraction buffer. The tubes were then bathed at 65°C and gently mixed until adequate homogenization. After centrifugation, the supernatants were mixed with an equal volume of phenol chloroform isoamyl alcohol (25:24:1) which were centrifuged at 12,000 rpm for 10 min. Chloroform isoamyl alcohol (24:1) was added to supernatants, followed by mixing and 10 min centrifugation at 12,000 rpm. Next, the supernatants were transferred into 1.5-mL tubes with isopropyl alcohol, mixed by inversion, and incubated at −20°C. After the precipitation and solution separated, the tubes were centrifuged at 12,000 rpm for 10 min and the precipitations were washed with 1 mL 75% ethanol twice. After drying the precipitation, ddH_2_O and 1ul of RNase were added. The mixtures were incubated at 37°C for 15 min.

### 2.4 Metagenomic analysis

#### 2.4.1 DNA library constructing, sequencing, and preprocessing

NEBNext^®^ UltraTM DNA Library Prep Kit for Illumina (NEB, USA) was used for DNA extraction to construct sequencing libraries, following the manufacturer’s protocols. The qualified library was sequenced on the Illumina NovaSeq PE150 platform at Wekemo Tech Co., Ltd. Shenzhen, China.

Raw sequenced libraries were processed at Wekmo Tech Co. using Kneaddata software. First, sequencing adaptors, amino acid bases with quality scores less than 20, and sequences shorter than 50 bp were trimmed based on the Trimmomatic algorithm (Bolger et al., 2014). To reduce host pollution, genes of soil bacteria origin were deleted by aligning the sequenced data with bacterial genes on Bowtie2 (Langmead and Salzberg, 2012). Finally, clean data were obtained.

#### 2.4.2 Taxonomy annotation

To understand the taxonomic distribution of the DNA library, kraken2 and a self-built database (Wekemo Tech Co.) were used to classify and annotate genes in the sample. Kraken2 that stores data of *k*-mer and its least common ancestor (LCA) matches the gene sequence with the most specific node of the taxonomic tree (Wood and Salzberg, 2014). After identifying the taxonomic distribution, Bracken was used to predict the actual relative abundance of species in the samples. By redistributing reads to species level through probability calculation, this method generated a species estimation of the dataset (Lu et al., 2017).

#### 2.4.3 Functional gene analysis

The clean sequences were compared with the protein database UniRed90 using HUMAnN3 software (Franzosa et al., 2018). After filtering out failed reads, the relative abundance of the functional genes was calculated using the TPM metric, which counts all matching reads in a fragment as one fragment count. This method calculated the proportion over an arbitrary total count, which is useful for comparing different samples (Puente-Sánchez et al., 2020; Zhao et al., 2021). Annotated genes were then sent to the ARDB database for ARG identification and to the KEGG database for host organism annotation (Liu and Pop, 2009; Kanehisa et al., 2017).

#### 2.4.4 Statistical analysis

Basic data analysis was performed on Microsoft Excel 2016 to obtain averages and standard deviations. One way ANOVA test carried out on Wekemo (Wekemo Tech Co. was used to analyze the statistical significance of changes caused by sulfamethoxazole. Any differences with a *p*-value lower than 0.05 were considered significant.

## 3 Results and discussions

### 3.1 Antibiotic impact on plant growth

**FIGURE 1:**
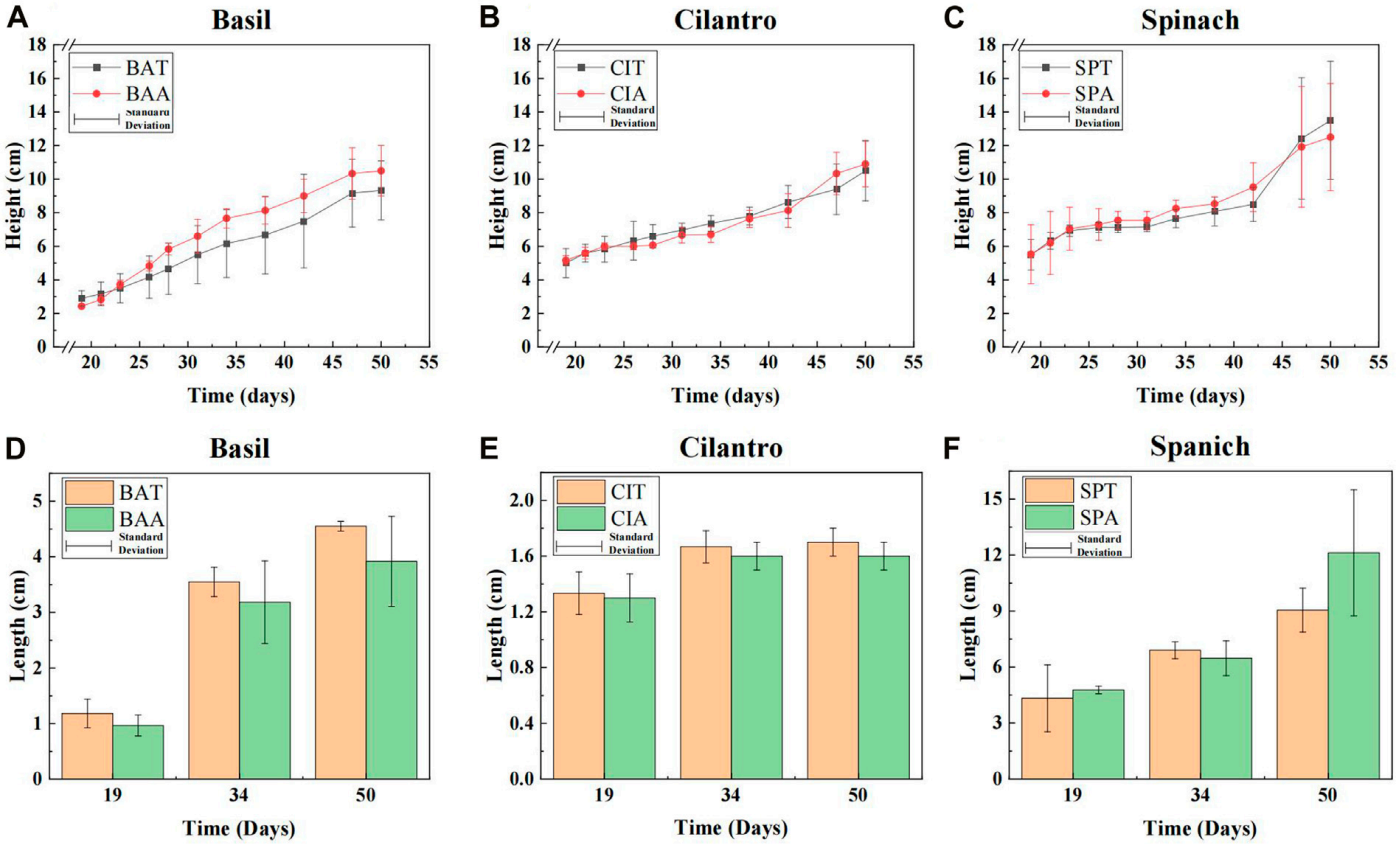
**(A–C)**: Stem heights of basil, cilantro and spinach. **(D–F)**: Leaf lengths of basil, cilantro, and spinach at the start, middle, and end of the measuring period.

Vegetable growth under SMX stress was accessed through two parameters visualized in Figure 1: stem height (graph a-c) and leaf length (graph d-f). At the start of the measuring period, all plants treated with SMX solution had similar lengths compared to those under normal conditions. A linear regression analysis showed that SMX solution improved basil growth on average by 0.05 cm/day and its final medium height (10.5 cm) surpassed the height under control conditions (9.3 cm) by 12.9%. However, SMX only changed the growth of cilantro and spinach by about 0.01 cm/day. A further significance analysis corresponded to the linear regression, revealing that 75 mg/L SMX solution did not pose significant impacts on basil, cilantro, and spinach growth (*p*-value >0.05 for all three plants).

Compared to the effect on stem growth, SMX had a clearer impact on leaf growth in all vegetables. Graph d-f in Figure 1 shows the leaf lengths at the start, middle, and end of the measuring period. Opposite to SMX’s facilitation in basil stem development, antibiotics led to smaller basil leaves throughout the measuring period. Its final medium length (3.92 cm) under SMX treatment was 13.8% shorter than the average leaf length (4.55 cm) of the control group. SMX also adversely affected the development of cilantro leaves with a final inhibition rate of 5.9%. Spinach leaf lengths were similar between the antibiotic group and the control group at the start and middle of the growth period. However, by the end of the period, Spinach leaves in the antibiotics group (12.13 cm) significantly outperformed the control group (9.05 cm) by 34.0% (*p*-value = 0.042 < 0.05).

Stem and leaf growth are indicators to assess the phytotoxicity of environmental pollutants (Mei et al., 2021). The data showed that SMX insignificantly promoted the basil stem growth. This result corresponded to Zhang et al.‘s study on pakchoi (*B. chinensis*) where SMX improved pakchoi height (Zhang et al., 2017). Interestingly, our findings showed the opposite of the general understanding that SMX adversely affects plant growth (Cheong et al., 2020). For instance, high SMX concentration in the soil could lead to a 44.7% decrease in tomato plant height and a 38.2% decrease in cucumber plant height. Similarly, Lv et al. found that exposure to 100 mg/L SMX solution resulted in a 28.13% decrease in strawberry (*Fragaria ananassa*) stem weight (Lv et al., 2021). This dichotomous effect of SMX between our results and the general knowledge could be explained by the plant’s hormetic response to antibiotics. Researchers found that while high concentrations of antibiotics tend to inhibit plant growth, low concentrations actually facilitate stem development (Migliore et al., 2000). This phenomenon was supported by recent studies. The low dosages of SMX led to longer stems in both pakchoi and strawberry, while higher concentrations reduced the stem length (Zhang et al., 2017; Lv et al., 2021). Therefore, it is possible that under SMX stress basil exhibited an insignificant height increase, while cilantro and spinach were not affected at all.

SMX impacted the leaf differently compared to its effect on plant height. SMX’s inhibition effect on basil and cilantro leaves could be due to the antibiotic’s higher concentration in leaves than stems. Although the sulfamethoxazole uptake mechanism is not fully understood, the majority of studies have pointed to passive diffusion as the main pathway (Franklin et al., 2016). Thus, the transpiration stream plays a crucial role in SMX’s translocation. Since stems are the main antibiotic channels rather than reservoirs, stems do not retain as many antibiotics as leaves (Liu et al., 2013). Several studies have shown that leaves accumulate more antibiotics than stems (Herklotz et al., 2010; Liu et al., 2013; Zhao et al., 2019). Therefore, basil and cilantro leaves could accumulate higher antibiotic concentrations than stems, which inhibits leaf development. On the other hand, spinach tends to accumulate fewer antibiotics in its tissues than other plants (Kodešová et al., 2019). Thus, SMX could exist in spinach leaves at a relatively lower concentration just enough to stimulate leaf growth.

The results showed that plants had different antibiotic tolerance. While SMX led to longer basil stems and larger spinach leaves, it suppressed cilantro’s growth. However, SMX’s impact on plant growth was overall insignificant, which suggested that vegetable stem and length lengths were not reliable indicators of SMX presence in the soil. Despite these insignificant impacts, SMX could still noticeably affect rhizobacteria diversity. Therefore, a further investigation into the microbial community under SMX stress is needed.

### 3.2 Analysis of microbial diversity

Raw sequence libraries were constructed based on the soil sample from eight pots. The number of raw reads ranged from 22884329 to 33657674 (avg. 26714901). Low-quality or short sequences were filtered out with the Trimmomatic algorithm, followed by the removal of host gene. As a result, 21521875 to 32085255 clean reads (avg. 25269515) were obtained for each group. A comparison with kraken2 and a self-built database (Wekemo Tech Co.) revealed the taxonomic distribution of each sample.

Alpha diversity indices, calculating each sample based on 5329 identified operational taxonomic units (OTUs), are shown in Table 2. Both the abundance-based coverage estimator (ace) and chao1 assessed the species richness. Compared to ace, chao1 gave more weight to the species of low abundance (Bo-Ra et al., 2017). Higher ace and chao1 values were observed for soil, basil, and cilantro treated with SMX. This result suggested that the presence of SMX in the sample increased the species richness except for spinach. Shannon and Simpson’s indices reflected bacterial diversity through richness and evenness. While Shannon emphasized more on bacterial species richness, Simpson focused on evenness (Suturina et al., 2022). As both Shannon and Simpson indices were generally higher for SMX groups than control, SMX in this experiment increased bacterial richness and evenness in all samples.

**TABLE 2:** Abundance indices of microbial community composition in each group.

	SOA	SOT	BAA	BAT	CIA	CIT	SPA	SPT
ace	2947.77	2579.23	3003.68	2544.86	2877.54	2737.11	2588.60	3119.70
chao1	2989.75	2614.88	3009.34	2588.40	2907.49	2760.09	2619.62	3161.36
Shannon	5.62	5.53	5.97	5.93	5.89	5.96	5.98	5.40
Simpson	0.88	0.88	0.92	0.89	0.91	0.89	0.91	0.90

**FIGURE 2:**
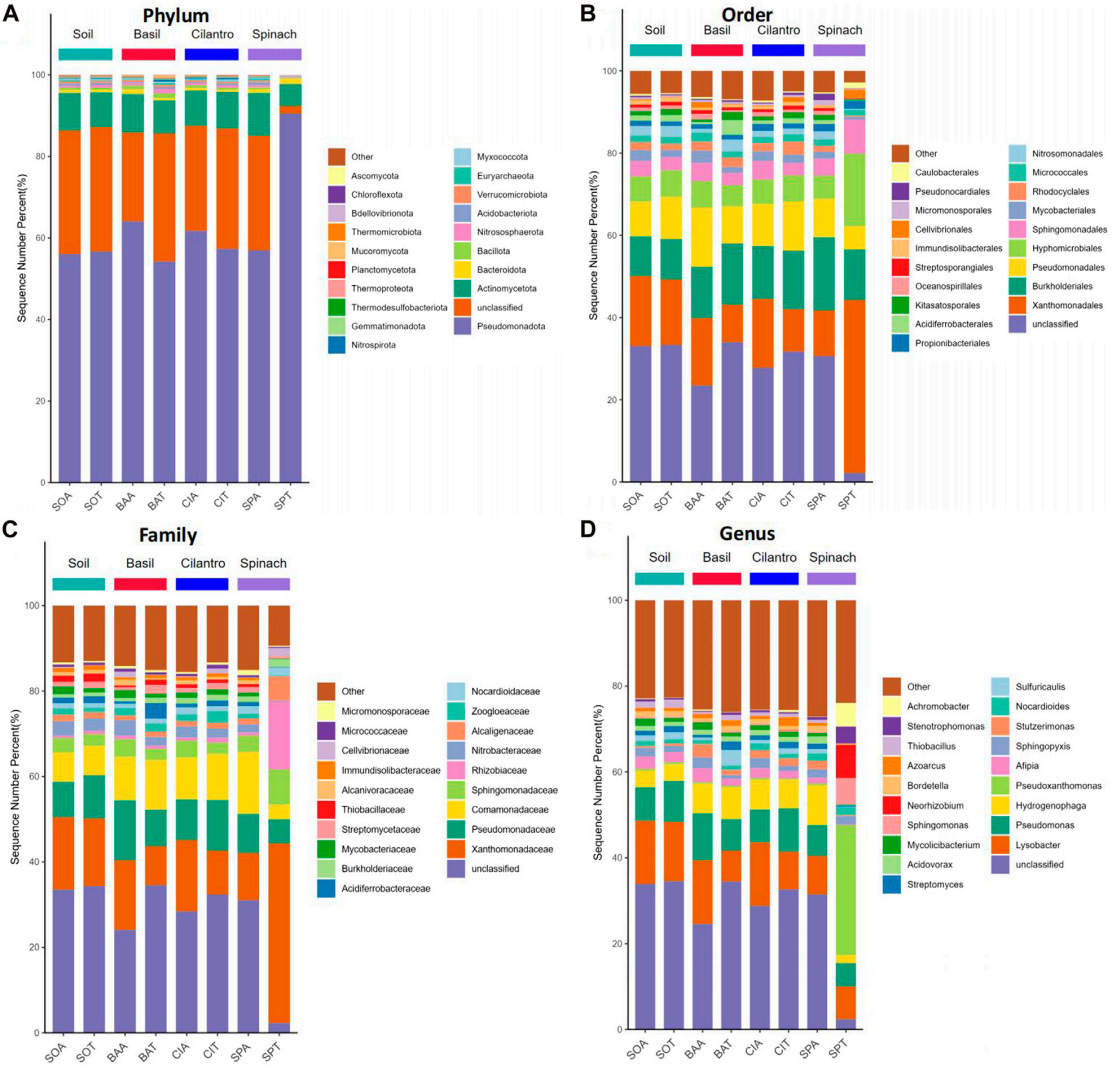
Relative bacterial abundance of eight groups at phylum, order, family, and genus level. **(A)** Relative bacterial abundances of the eight groups at the phylum level. **(B)** Relative bacterial abundances of the eight groups at the order level. **(C)** Relative bacterial abundances of the eight groups at the family level. **(D)** Relative bacterial abundances of the eight groups at the genus level.

The microbial composition at phylum, order, family, and genus levels are presented in Figure 2. SMX changed microbial composition in pots with the vegetables. At the phylum level, *Pseudomonadota* was identified as the dominant phylum for all samples, followed by *Actinomycetota*. *Pseudomonadota*, better known as proteobacteria, is the most prevalent bacterial phylum in the environment (Leão et al., 2023; Selmani et al., 2023). This phylum includes many rhizobacteria associated with plant growth promoting activities, including nitrogen fixation, phosphorus uptake, and antifungal effect (Ramakrishna et al., 2019; Gómez-Godínez et al., 2023). At order rank, *Xanthomonadales*, *Burkholderiales*, and *Pseudomonadales* are the top three most dominant orders all of which belong to the phylum *Psedomonadota*. SMX increased the richness of *Xanthomonadales* in BAA (16.4%) and CIA (16.8%), compared to *Xanthomonadales* proportion in BAT (9.2%) and CIT (10.3%). At the family level, *Xanthomonadacae*, under order *Xanthomonadales*, was the most dominant family. It existed in a larger abundance in BAA and CIA than BAT and CIT. Although SPA had a similar soil profile to other plants, SPT altered the microbial composition significantly. *Xanthomonadacae* took up 42.1% in SPT, compared to the average abundance of *Xanthomonadacae* in the rest of the samples (13.8%). Spinach also facilitated the proliferation of *Rhizobiaceae* (15.9%), a family under *Hyphomicrobiales*, which is a symbiotic nitrogen-fixing bacteria (Ormeño-Orrillo et al., 2015). Gómez et al. found that *Rhizobium* could colonize spinach roots and promote root, shoot, and leaf growth (Jimenez Gómez et al., 2016).

At the genus level, the effect of antibiotics was registered across all plant types. *Lysobacter* under the family *Xanthomonadacae* was the dominant genus. Its abundance in basil, cilantro, and spinach with SMX (14.9%, 14.8%, 9.0% respectively) was consistently higher than in samples treated with water (7.2%, 8.8%, 7.7%). *Lysobacter* protects plants through the release of antimicrobial compounds and facilitates the degradation of macromolecules with extracellular enzymes (Gómez Expósito et al., 2015; Brescia et al., 2020). Its high concentration in sulfamethoxazole-contaminated soil could be explained by plants’ need for antibiotic degradation. *Pseudomonas* and *Hydrogenophaga* were the second and third most abundant genus. While *Pseudomonas* promotes plant growth and phytopathogen control, *Hydrogenophaga* modifies pollutants through enzymic activities and transforms them into less toxic substances (Sah et al., 2021; Majeed et al., 2022). SMX did not have noticeable impacts on the abundance of *Pseudomonas* and *Hydrogenophaga*. Three genera emerged from SPT, including *Pseudoxanthomonas*, *Sphingomonas,* and *Neorhizobium*. *Peseudoxanthomonas* and *Neorhizobium* facilitate plant growth through molecule degradation and nitrogen-fixing respectively. *Sphingomonas* increase plant’s stress tolerance and production of phytohormone (Sah et al., 2021; Lombardino et al., 2022; Marzba et al., 2022).

**FIGURE 3:**
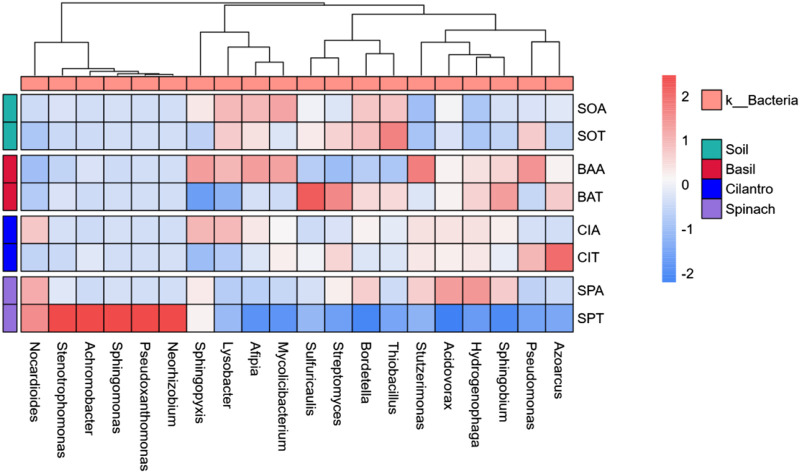
Heatmap comparing bacterial relative abundance across eight groups at the genus level.

From the heat map in Figure 3, SPA had high abundances of *Sphigobium*, *Hydrogenphaga*, *Acidvorax*, *Stutzerimonas*, and *Bordetella*, which could induce spinach’s larger leaf length. On the other hand, SMX led to decreases in the abundance of *Streptomyces* and *Sulfuricaulis* in the basil and cilantro rhizosphere. *Streptomyces* aids the plant with nitrogen, phosphorus, and potassium uptake while serving as a biocontrol agent against plant pathogens (Amaresan et al., 2018). *Sulfuricaulis* plays the role of a sulfur-oxidizer. The decrease in *Streptomyces* and *Sulfuricaulis* under SMX stress might eventually lead to smaller basil and cilantro leaves.

Overall, SMX impacted the bacterial composition and increased the diversity in all samples of this experiment. Past studies have shown that antibiotics altered the bacteria diversity through the elimination of sensitive bacteria and the outgrowth of resistant bacteria (Cycoń et al., 2019). Corresponding to the study by Reichel et al., *Pseudomonas* were insignificantly impacted by SMX, possibly because of the prevalence of resistance genes in *Pseudomonas* (Reichel et al., 2014). SMX’s impact on bacterial composition could contribute to the changes in plant growth. For instance, a decrease in the abundance of *Streptomyces* and *Sulfuricaulis* could have led to smaller basil and cilantro leaves, while the increase in *Sphigobium*, *Hydrogenphaga*, *Acidvorax*, *Stutzerimonas*, *Bordetella* might facilitate spinach leaf growth. Although the analysis of microbial diversity demonstrated SMX’s impact on soil bacteria, an examination of ARGs was still needed to study the correlation between antibiotics and ARB.

### 3.3 Functional gene host identification and interbacterial relationships

**FIGURE 4:**
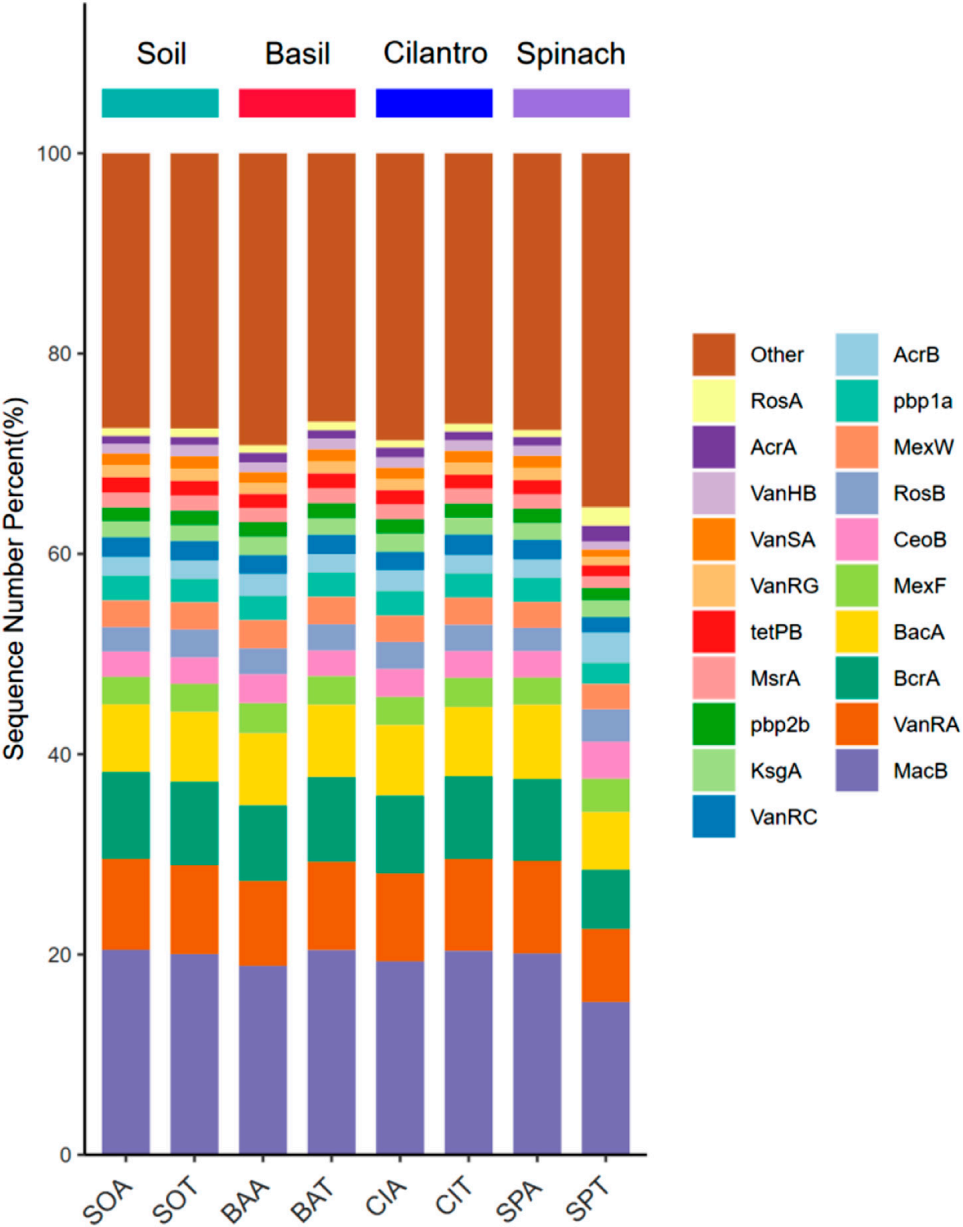
Abundance of ARGs in each sample.

The cleaned sequence library was compared with the protein database UniRef 90. ARGs in each sample were identified through the ARBD database. According to Figure 4, *MacB* was the most abundant gene in every rhizosphere sample (average 19.4%). This gene encodes for an ATP-binding cassette (ABC) transporter that exports antibiotics specifically macrolide through mechanotransmission (Greene et al., 2018). Among the top 10 genes, 7 genes encoded for antibiotics efflux pump (*MacB*, *BcrA*, *MexF*, *CeoB*, *MexW*, *RosB*, *AcrB*), and 3 genes encoded for antibiotics target alternation (*VanRA*, *BacA*, *Pbp1a*) (Charlebois et al., 2012; Stogios and Savchenko, 2020; Fauzia et al., 2023). The presence of SMX spiked the amount of *Sul1* on average by 108% and increased the amount of *Sul2* by 33.7% (*p*-value = 0.006). Both *Sul1* and *Sul2* protect the bacteria through antibiotic target alternation by encoding for dihydropteroate synthase (DHPS) with low affinity to sulfamethoxazole. This discovery supported the fact that antibiotics could establish selective pressure that led to the proliferation of specific ARGs (Skandalis et al., 2021).

**FIGURE 5:**
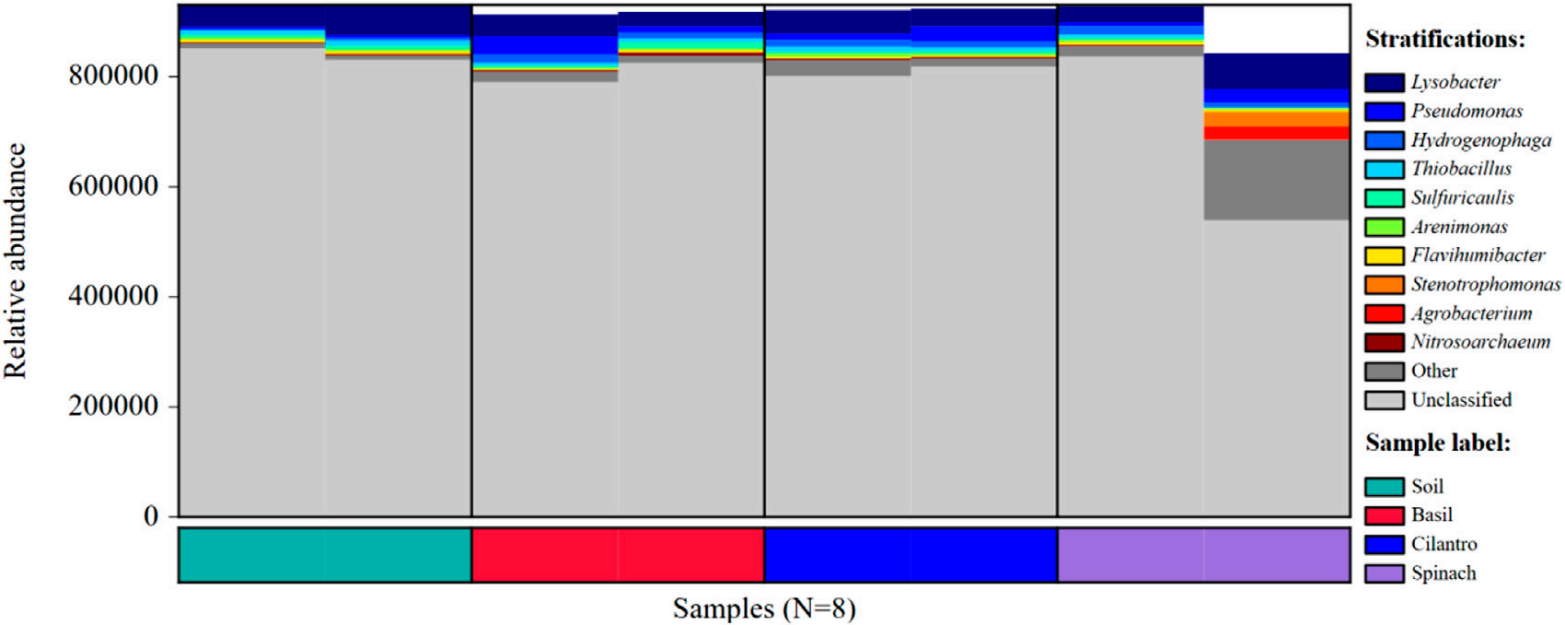
Heatmap of key functional gene hosts in each sample.

**FIGURE 6:**
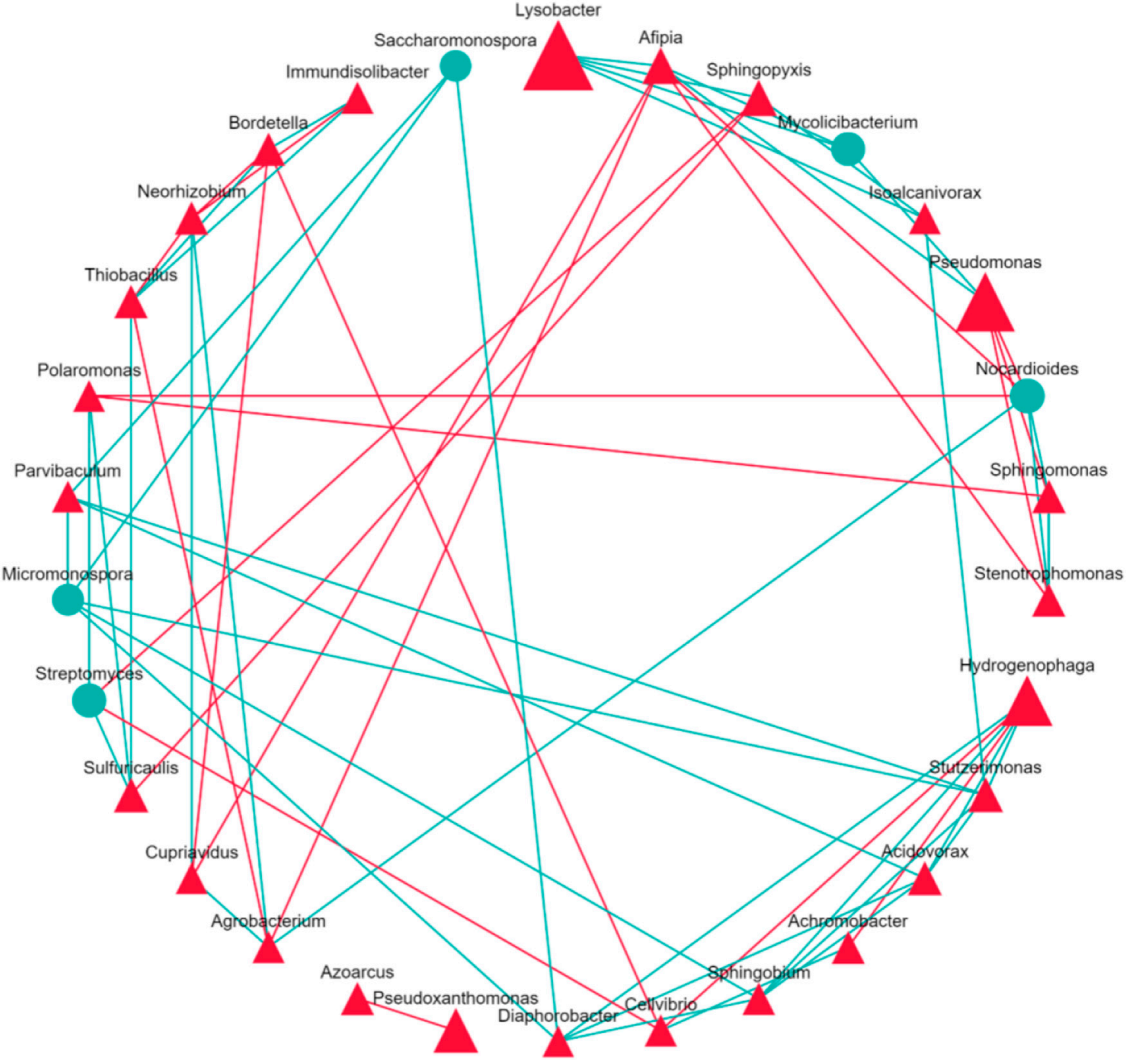
Correlation between bacteria genera. The triangle symbolizes phylum Pseudomonadota, circle symbolizes the phylum Actinomycetota. The green line for positive and the red line for negative correlation.

KEGG orthology database identified host bacteria and annotated functional genes similar to known resistance genes (Martínez et al., 2015). Figure 5 reveals the concentration of functional genes among bacterial genera. Due to its greatest abundance, *Lysobacter* hosted most functional genes. Among the top 200 species, genus *Pseudomonas* held 13,778 reads on average, while *Hydrogenophaga* contained 84,529 reads. *Pseudomonas*’s abundance was only 3.6% higher than *Hydrogenophaga*’s, but *Pseudomonas* held 62.4% more functional genes. This finding revealed that *Pseudomonas* tend to host more functional genes than *Hydrogenphaga*. Correspondent to our discovery, Ashy et al. also concluded that *Pseudomonas* was a key host for viral genes and antibiotic resistance genes in the rhizosphere of moringa (*Moringa oleifera*) (Ashy et al., 2023). In the soil sample of spinach treated with water, *Stenotrophomonas* and *Agrobacterium* were not abundant genera, but hosted relatively large amounts of functional genes. The presence of those functional gene hosts, namely, *Pseudomonas*, *Stenotrophomonas*, and *Agrobacterium*, could facilitate the proliferation of ARGs. Figure 6 visualizes the interactions among bacterial genera. *Shingomonas* and *Alfipia* are two common bacteria that did not carry many functional genes but hindered other key hosts. *Shingomonas* competed with *Pseudomonas*, while *Alfipia* limited the growth of *Stenotrophomonas* and *Agrobacterium*. Therefore, it would be potentially beneficial to monitor and facilitate the growth of *Shingomonas* and *Alfipia* to outcompete functional gene hosts.

## 4 Conclusion

This study analyzed sulfamethoxazole’s impact on vegetable growth, soil bacteria composition, and ARG profile. In the experiment, SMX promoted the development of spinach leaves and basil stems, but had no significant impact on cilantro growth. A further study into the rhizosphere bacterial composition showed an increased microbial diversity under SMX stress. Most of the soil bacteria identified promoted plant growth through nutrient uptake and antimicrobial defense. SMX eliminated sensitive bacteria and increased the proportions of resistant species. These shifts in the bacterial community could contribute to the observed changes in plant growth. SMX also led to the proliferation of the *Sul1* and *Sul2* genes. *Pseudomonas*, *Stenotrophomonas,* and *Agrobacterium* were identified as key virulent gene hosts. By analyzing bacterial interactions, this study recommended promoting the growth of *Shingomonas* and *Alfipia* in the soil to outcompete the resistance gene hosts, which could reduce the amount of ARGs. However, future investigation is required to verify and consolidate the method.

## Data Availability

The datasets presented in this study can be found in online repositories. The names of the repository/repositories and accession number(s) can be found a the following link: https://doi.org/10.6084/m9.figshare.25115306.v1.
